# MUC5AC concentrations in lung lavage fluids are associated with acute lung injury after cardiac surgery

**DOI:** 10.1186/s12931-024-02747-9

**Published:** 2024-03-07

**Authors:** Judith van Paassen, Pieter S. Hiemstra, Abraham C. van der Linden, Evert de Jonge, Jaap Jan Zwaginga, Robert J.M. Klautz, M. Sesmu Arbous

**Affiliations:** 1 Department of Intensive Care, Leiden University Medical Center, Albinusdreef 2, Leiden, B4-57, 2333 ZA the Netherlands; 2 Department of Pulmonology, Leiden University Medical Centre, Leiden, the Netherlands; 3 Center for Clinical Transfusion Research, Sanquin Research, Leiden, the Netherlands; 4 Department of Hematology, Leiden University Medical Center, Leiden, the Netherlands; 5 Department of Thoracic Surgery, Leiden University Medical Center, Leiden, the Netherlands; 6 Department of Clinical Epidemiology, Leiden University Medical Center, Leiden, the Netherlands

**Keywords:** Cardiac surgery, ARDS, Pulmonary biomarkers, Mucins, Perioperative care, Intensive care

## Abstract

Heart surgery may be complicated by acute lung injury and adult respiratory distress syndrome. Expression and release of mucins MUC5AC and MUC5B in the lungs has been reported to be increased in acute lung injury. The aim of our study was to [1] investigate the perioperative changes of MUC5AC, MUC5B and other biomarkers in mini-bronchoalveolar lavage (minBAL), and [2] relate these to clinical outcomes after cardiac surgery. In this prospective cohort study in 49 adult cardiac surgery patients pre- and post-surgery non-fiberscopic miniBAL fluids were analysed for MUC5AC, MUC5B, IL-8, human neutrophil elastase, and neutrophils. All measured biomarkers increased after surgery. Perioperative MUC5AC-change showed a significant negative association with postoperative P/F ratio (*p* = 0.018), and a positive association with ICU stay (*p* = 0.027). In conclusion, development of lung injury after cardiac surgery and prolonged ICU stay are associated with an early increase of MUC5AC as detected in mini-BAL.

**Supplementary Information:**

The online version contains supplementary material available at 10.1186/s12931-024-02747-9.

## Background

Development of acute lung injury, sometimes even overt acute respiratory distress syndrome (ARDS), is a complication following cardiac surgery that is reported in 1–8% of patients [1]. ARDS is associated with a complicated postoperative course, [2] high mortality (50–90%) [1–3], and significant long term physical and psychological sequelae [4]. 

Activation of several inflammatory pathways in the pathogenesis of acute lung injury and ARDS following cardiac surgery have been studied extensively, including the roles of pathogen- and damage associated molecular patterns, pattern recognition receptors, release of pro- and anti-inflammatory cytokines, complement activation and activation of neutrophils and platelets [5, 6]. Human Neutrophil Elastase (HNE), released by degranulating neutrophils, may cause local tissue injury, leading to capillary leakage and accumulation of protein-rich exudate [1, 7]. HNE is a secretagogue for mucus-producing goblet cells in the airway epithelium and submucosal glands, and increases the expression of mucin proteins, including MUC5AC and MUC5B, that are implicated in the pathogenesis of asthma, chronic obstructive pulmonary disease (COPD) and cystic fibrosis [7, 8] Up to now, the role of MUC5AC and MUC5B in development of acute lung injury after cardiac surgery is unknown.

In this study, we hypothesized that mucin production may contribute to development of acute lung injury after cardiac surgery. Therefore, we aimed to investigate the perioperative changes of MUC5AC and MUC5B and other biomarkers in relation to postoperative acute lung injury and other clinical outcomes.

## Main text

### Methods

An explorative prospective cohort study was performed in the intensive care unit (ICU) of the Leiden University Medical Center (LUMC) in 49 patients after elective cardiac surgery. The study was approved by the medical ethical committee (protocol P117-11), and registered under Clinical Registration number: ICTRP: NTR 5314, 26-05-2015. Exclusion criteria were inability to sign informed consent, and preoperative corticosteroid use. The standardized peri-operative care path is summarized in the supplementary material. Non-fiberscopic mini-bronchoalveolar lavages (miniBAL) were performed at two time points: pre-surgery after intubation (T1), and at ICU arrival (T2). Samples were processed in the laboratory immediately upon collection. After mucolysis and filtering, the cells and debris were separated by centrifugation. The pelleted cells were resuspended and manual differential cell counts of eosinophils, neutrophils, lymphocytes, macrophages, and epithelial cells were performed. In the remaining supernatant the levels of soluble HNE, IL-8, MUC5AC and MUC5B protein were determined by enzyme-linked immunosorbent assay (ELISA) and dot blot-based immunochemical assays (see also supplementary material) [9, 10]. The primary endpoint of this study was to investigate perioperative change of MUC5AC, MUC5B, IL8, leukocytes, and HNE in respiratory secretions in relation with the development of acute lung injury (measured as PaO2/FiO2 (P/F) ratio and other clinical outcomes (ICU stay and hospital stay).

### Statistical analysis

Correlations between perioperative biomarkers were tested using Pearson’s correlations. If a perioperative change could not be calculated due to missing values, or if a P/F ratio was not available that patient was excluded from that specific analysis. For biomarkers, differences at a p-value < 0.05 were considered significant. The statistical analyses were conducted using the SPSS (Statistical Package for the Social Sciences), release 25.0 (SPSS Inc., Chicago).

### Results

In supplementary Table 1 patient characteristics and major clinical outcomes are shown. Patients were predominantly male, and the majority had a history of myocardial infarction, diabetes, smoking, and hypertension. Five patients had a moderately to severely impaired left ventricular function. The surgical procedures varied from coronary artery bypass grafting (CABG) to more complex combined valve surgery, pericardiectomy, left-ventricular reconstructive surgery, and heart failure surgery. Seventeen patients (30%) developed acute lung injury fulfilling the Berlin criteria post-surgery, of whom two (4%) severe ARDS [11]. One patient did not survive due to an exacerbation of underlying inflammatory lung disease.

**Fig. 1:**
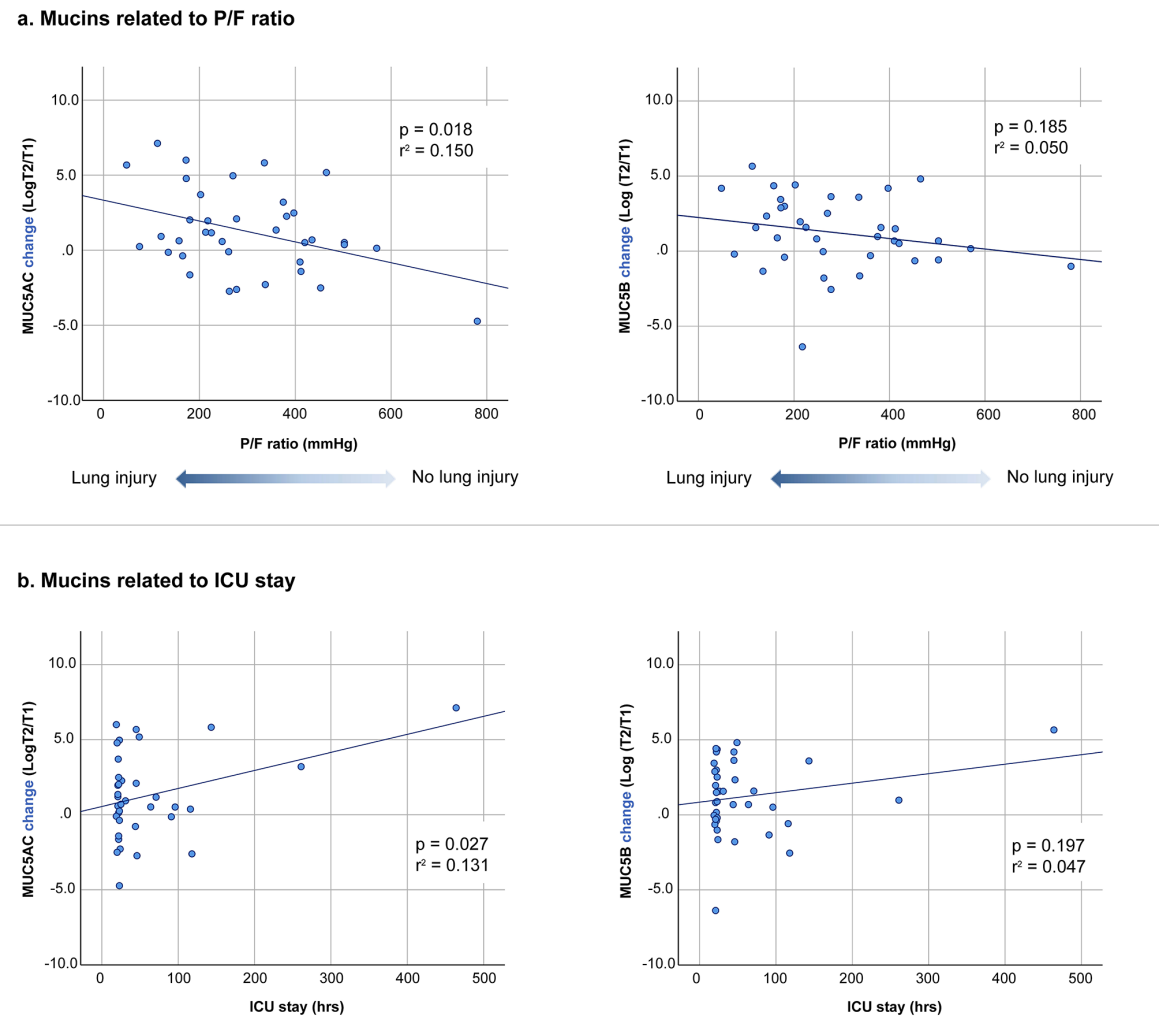
Peri-operative change in Mucins MUC5AC and MUC5B related to lung injury (P/F ratio) and ICU stay. T1 = pre-operative timepoint; T2 = ICU arrival timepoint; P/F ratio = PaO2/FiO2 ratio

Absolute values for the different biomarkers at T1 and T2 are given in supplementary Table 2. Neutrophils, IL8, HNE, MUC5AC and MUC5B all increased peri-operatively. For all biomarkers, the degree of perioperative change, measured as Log T2/T1, was tested for correlation with the P/F ratio as a continuous variable. A significant (*p* = 0.018) negative correlation was found between MUC5AC and P/F ratio (Fig. 1a). Furthermore, a positive correlation of the perioperative MUC5AC change with ICU length of stay was found (*p* = 0.027) (Fig. 1b). No significant association with other clinical outcome parameters was observed for any of the other biomarkers (supplementary Table 3).

### Discussion

In this study we show that concentrations of mucins in airway lavage fluid are increased peri-operatively from induction of anaesthesia until admission to the ICU, and that the increase in MUC5AC is related to the severity of acute lung injury and the length of ICU stay. Not only mucins, but in fact all measured biomarkers increased peri-operatively. Thus, the increase of mucins may simply be one of many reflections of the activation of inflammation that takes place after cardiac surgery [6]. However, the magnitude of increase is five- to tenfold for MUC5B and MUC5AC and thereby much more than the increase of other biomarkers. Also, MUC5AC was the only biomarker for which the perioperative increase was significantly associated with relevant clinical endpoints. Thus, we cannot rule out that mucins have an etiologic role in the development of acute lung injury and ARDS after heart surgery.

Our results are in line with one earlier study in children after cardiac surgery with cardiopulmonary bypass. Children with respiratory complications showed significantly higher MUC5AC levels in lavage fluid after surgery than did children without respiratory complications and the increase of total mucin cardiopulmonary bypass showed positive correlation with alveolo-arterial oxygen difference [12]. 

Mucins are major glycoprotein components of mucus and are important in pulmonary mucosal defence. MUC5AC is produced in the superficial mucosa and MUC5B primarily in the submucosal glands [13]. In normal airways, mucins cover the epithelial surface of the respiratory tract, and mucin production is maintained at a relatively low level to promote mucociliary clearance of inhaled and trapped substances. In pathologic conditions, excessive mucus production limits mucocilliary clearance, whereas mucus accumulation may lead to mucus plugging and airway obstruction, ultimately impairing gas exchange [14]. In critically ill patients with acute lung injury, MUC5AC levels in bronchoalveolar fluid were more than 58-fold increased [15]. The concomitant elevation of the secretagogue HNE and the short time interval after start of surgery suggests a role for hypersecretion of mucin by already present goblet cells rather than upregulation of the number of goblet cells. The strong association between MUC5AC and acute lung injury does not necessarily imply that mucins play a direct etiologic role in acute lung injury. Alternatively, increased mucin expression could also be just a reflection of the proinflammatory state without a specific causal role.

*In conclusion*, we show a marked increase in the concentrations of MUC5A and MUC5B in bronchoalveolar fluid of patients after heart surgery and a significant association between the increase of MUC5A and the severity of acute lung injury and length of ICU stay. To better understand the importance of mucins, it would be interesting to study the influence of specific inhibition of MUC5AC in situations leading to acute lung injury.

### Electronic supplementary material

Below is the link to the electronic supplementary material.

Supplementary Material 1

## Data Availability

Access to data used for this publication has been limited to the principle investigator and collaborators. Before the data can be shared, an additional research application must be submitted to and approved by our medical ethics committee.

## Declarations

### Trial registration data

The study was registered under Clinical Registration number: ICTRP: NTR 5314, 26-05-2015.

## Abbreviations

ARDS: Acute respiratory distress syndrome
IL: Interleukin
HNE: Human neutrophil elastase
P/F ratio: PaO_2_/FiO_2_ ratio
MUC: Mucin
ICU: Intensive care unit
SIRS: Systemic inflammatory response syndrome
PAMP: Pathogen associated molecular patterns
DAMP: Damage associated molecular patterns
NETs: Neutrophil extracellular traps
COPD: Chronic obstructive pulmonary disease
AU/ml: Arbitrary units/ml
