# Supplementary material for: MUC5AC concentrations in lung lavage fluids are associated with acute lung injury after cardiac surgery
Source: Respir Res. 2024 Mar 7;25:117. doi: 10.1186/s12931-024-02747-9 (PMC10921709; doi:10.1186/s12931-024-02747-9)
Supplement: Supplementary file 1 — Supplementary Material 1 [file 12931_2024_2747_MOESM1_ESM.docx]

**Supplemental material**

**Inflammatory biomarkers in the respiratory tract and lung injury in cardiac surgery patients**

**Contents**

Peri-operative management

Biochemical Assays miniBAL fluid

Table S1. Patient characteristics

Table S2. Values for neutrophils, IL-8, HNE, MUC5A and MUC5B pre-operatively after induction of anaesthesia (T1) and at ICU-admission (T2)

Table S3. Correlation between increase of biomarkers measured in miniBAL as concentration at T2 divided by concentration at T1 (T2/T1), and clinical outcomes.

References

**Peri-operative Management**

Pre-operative screening: All patients visited the pre-operative outpatient clinic. After admittance, all patients were assessed by an anesthesiologist and a thoracic surgeon on the day before surgery. In case of suspicion of an active infection e.g. on the basis of respiratory symptoms or elevated temperature, surgery was postponed.

Anaesthesia: Standard premedication and induction medication were followed by total intravenous anaesthesia. All patients received a central venous and an arterial catheter, standard surgical antibiotic prophylaxis, and tranexamic acid (loading dose 15 mg/kg anticipating a perfusor 5 mg/kg/hr) to minimise perioperative blood loss. Mechanical ventilation consisted of a standard protocol with low pressure and low tidal volume during CPB to prevent atelectasis of the lung. Inotropic and vasoactive agents were administered on indication. Applied transfusion triggers were congruent with the national guidelines(1) and corticosteroids were only administered in pre-defined situations, e.g. chronic corticosteroid-use, severe inflammatory response with hemodynamic instability during surgery.

Cardiopulmonary Bypass: A heart-lung machine (S3; Sorin Group) with a centrifugal blood pump (Revolution; Sorin Group) and a hollow fibre membrane oxygenator (Maquet Quadrox-I or Terumo FX15) was used. The circuit was primed with hydroxyethyl starch 130 (Voluven 6%; Fresenius Kabi Norge AS), Mannitol 15%, Ringer’s solution and Heparin. Tubing was coated with bio-inert heparin-free polymers (SAFELINE; MAQUET Holding BV & Co KG). Intermittent warm antegrade blood cardioplegia was applied. Anticoagulant therapy consisted of heparin (target Activated clotting time > 400s) which was antagonised with protamine sulphate at the end of the procedure. The patient’s core temperature was maintained at 34oC to 36oC, except for aortic surgery where active cooling (up to 20oC) was used to prevent brain ischemia.

Postoperative care: Patients were treated according to a so called “fast-track” protocol (2) In short: when hemodynamically stable and with a core temperature of > 36 °C, patients were extubated when the following criteria were met: 1) Ramsay score 2-3, (3) 2) Arterial pO2 > 9 kPa and FiO2 40%, 3) pH > 7.30 and 4) decreasing thoracic drain production < 50-100ml/hour. Patients were discharged from the ICU on the first postoperative day, unless the patient’s hemodynamic and respiratory status required prolonged ICU stay. Hemodynamic monitoring was performed by an arterial and central venous catheter and, on indication, with a pulmonary artery catheter. In patients with low cardiac output, dobutamin, enoximone/milrinone, and noradrenaline were administered as inotropic and vasoactive agents.

**Biochemical Assays miniBAL fluid**

*Sample collection:* In intubated patients, non-fiberscopic mini-bronchoalveolar lavages (miniBAL) were performed at two time points: pre-surgery after intubation (T1), and at ICU arrival (T2). Via the endotracheal tube a CombiCath® catheter was introduced and 10 ml NaCL 0.9% was instilled and aspirated again. Dwelling time was short because otherwise there was not enough yield with the small volumes used. The catheter was advanced to wedge. It is assumed that material from the airways of approximately 1.3 mm in diameter, i.e. the diameter of the catheter, was obtained.

*Laboratory analyses:* Samples were processed in the laboratory immediately upon collection. After mucolysis using Sputolysin Reagent [DTT] (Calbiochem, cat nr. 560000) and filtering (using a 100-micrometre filter) the cells and debris were separated by centrifugation for 10 min, at 1500 rpm and room temperature.

*Cell counts and differentiation:* The pelleted cells were resuspended in phosphate buffered saline (PBS) containing 1% (wt/vol) human serum albumin (HSA). To enable optimal cell differentials, the concentration was adjusted to a concentration of 0.2 x 106 cells/ml. The cytospins were stained with Quick-diff (Dade Int. Inc., Deerfield, IL, USA) and manual differential cell counts of eosinophils, neutrophils, lymphocytes, macrophages, and epithelial cells were performed. From each miniBAL sample, two slides were prepared and stained. In each slide, at least 100 nucleated cells were counted manually and expressed as a percentage of the total number of nucleated cells. Mean values of these percentages were used in the analyses. The remaining supernatant was collected and stored at minus 80oC and analysed later in one reagent batch to limit inter-assay variation.

*Biomarkers:* For Human neutrophil elastase HNE a sandwich ELISA technique was used. Briefly, microwells (Immulon 4; Dynatech Laboratories, Chantilly, VA) were coated overnight at room temperature with polyclonal rabbit anti-human elastase IgG (HNE). Both standard serial dilutions of elastase purified from purulent sputum [28] and samples were diluted in PBS containing 0.05% (vol/vol) Tween 20 and 1% (vol/vol) heat-inactivated newborn calf serum and incubated for 1 h at 37°C. Bound elastase was detected using biotinylated rabbit anti-human elastase IgG, followed by peroxidase-conjugated streptavidin, which were both incubated for 1 h at 37°C. The lower detection limit of the assay was < 0.4 ng/mL. Cross-reactivities of this ELISA with highly purified preparations of the PMNL proteinases, cathepsin G (purified from purulent sputum), and proteinase 3 (gift from M. Daha), were < 0.3% and < 0.01 % respectively.(4)

For IL-8: The levels of IL-8 were determined in the supernatant using enzyme-linked immunosorbent assay (ELISA) techniques. For IL-8, a commercial kit using mouse anti-human IL-8 antibodies wase used (CLB; Amsterdam, the Netherlands).

For Mucin 5AC and Mucin 5B protein expression, cells were lysed in RIPA buffer according to the manufacturer's instruction (Thermo Fisher Scientific, Breda, The Netherlands). Lysate was diluted in bicarbonate coating buffer without azide and incubated in a NUNC maxisorp ELISA plate (Thermo Fisher Scientific) at 37°C until dry. Plates were washed and nonspecific binding sites were blocked with PBS/2% (w/v) BSA (Sigma-Aldrich Chemie BV, Zwijndrecht, The Netherlands) for 2 h at room temperature, followed by 2 h incubation with mouse anti-MUC5AC and anti MUC5B (1:200; 45M1; Thermo Fisher Scientific) in PBS/0.05% Tween-20 (v/v) (Sigma-Aldrich) at room temperature. Next, plates were washed with PBS/0.05% Tween-20 and incubated for 1 h with goat anti-mouse HRP (1:2000, Dako Denmark A/S, Glostrup, Denmark) at room temperature. Plates were developed using tetramethylbenzidine-hydrogen peroxidase solution and the reaction was stopped with 2.5 mol/L H2SO4. Absorbance was measured at 450 nm using a Microplate reader (iMark; Bio-Rad Laboratories, Hercules, CA) and Microplate Manager Software (version 6.3, Bio-Rad).(5)

**Table S1. Patient Characteristics and Outcome (n=49)**

| **Demographic parameters**  Age (yr) (mean, SD)  Gender (male) (n, %)  BMI (kg/m^2^) (mean, SD) | 66.4 ± 10.2  28 (57)  26.3 ± 4.3 |
| --- | --- |
| **Other relevant clinical data (n, %)**  Myocardial infarction in history  Percutaneous Catheter Intervention in history  Thoracic surgery in history  Hypertension  Malignancy in history  Chronic kidney insufficiency  Chronic liver disease  Diabetes  COPD  Smoking  Pack years (mean, SD)  Forced Vital Capacity (%) (mean, SD)  FEV_1_/FVC (without beta2-agonist) (mean, SD) | 10 (20)  14 (29)  3 (6)  26 (53)  4 (8)  4 (8)  1 (2)  12 (25)  6 (12)  31(63)  23.1 + 14.9  3.7 + 1.1  74.2 + 9.9 |
| **Ante-Surgery performance state (n, %)**  ASA I   II  III  IV  LVEF good LVEF > 55%  Reasonable LVEF 40-55 %  Moderate LVEF 25-40%  Poor LVEF < 25%  Euroscore logistic (median, IQR) | 0 (0)  8 (16)  38 (78)  3 (6)  29 (59)  15 (31)  3 (6)  2 (4)  6 (5.6-6.4) |
| **Surgical parameters**  Surgical procedure  CABG  CABG + single valve  CABG + multiple valve  Single valve  Multiple valve  Thoracic Aorta surgery (+/- valve +/- CABG)  Other Surgical duration  Surgery (hrs) (median, IQR)  Cardiopulmonary bypass (hrs) (median, IQR)  Aorta Clamp time (min) (median, IQR)  **Intraoperative Steroid use (n,%)** | 18 (37)  4 (8)  1 (2)  5 (10)  4 (8)  12 (25)  5 (10)  6.5 (6.2-6.8)  184.0 (127.9-195.1)  127.0 (118-136)  9 (18) |
| **Outcomes**  ARDS According to Berlin Definition (n, %)  Mild  Moderate  Severe  Ventilation time (min) (median, IQR)  Length of ICU stay (hrs) (median, IQR)  Length of Hospital stay (days) (median, IQR)  30 days mortality (n, %) | 9 (18)  6 (12)  2 (4)  724.0 (145-1303)  23.7 (20.7-27.0)  8.0 (3-13)  1 (2) |

BMI = body mass index: PCI = percutaneous catheter intervention: COPD = chronic obstructive pulmonary disease:
ASA = American society of anesthesiologists score: LVEF = left ventricular ejection fraction:
CABG = Coronary Artery Bypass Grafting: ARDS = Adult respiratory distress syndrome: ICU = Intensive care unit:

**Table S2. Values for neutrophils, IL-8, HNE, MUC5A and MUC5B pre-operatively after induction of anaesthesia (T1) and at ICU-admission (T2)**

| **Biomarker** | **Preoperative (T1)** | | **ICU arrival (T2)** | |  |
| --- | --- | --- | --- | --- | --- |
| *(available samples)* | *Mean* | *SD* | *Mean* | *SD* | *Ratio T2/T1* |
| Neutrophil (%) (27) | 25.0 | (30.3) | 39.2 | (28.0) | 1.6 |
| IL-8 (pg/ml) (13) | 3521 | (17099) | 8629 | (25535) | 2.5 |
| HNE (39) (AU/ml) | 210.3 | (417.9) | 403.79 | (683.8) | 1.9 |
| MUC5B (AU/ml) (42) | 55.88 | (157.9) | 256.2 | (461.3) | 4.6 |
| MUC5AC (AU/ml) (43) | 132.47 | (345.2) | 1164.6 | (3271.7) | 8.8 |

*IL-8 = Interleukin 8; HNE = Human Neutrophil Elastase; MUC5B = Mucin 5B; MUC5AC = Mucin 5AC; AE/ml = Arbitrary Units/milliliter*

**Table S3. Correlation between increase of biomarkers measured in miniBAL as concentration at T2 divided by concentration at T1 (T2/T1), and clinical outcomes.**

|  | **CPB-time** | **Simple vs complex Surgery*** | **Mechanical ventilation time** | **P/F ratio (mmHg)** | **ICU stay** | **Hospital stay** |
| --- | --- | --- | --- | --- | --- | --- |
| Neutrophil (%) | 0.048 (p=0.836) | 0.016 (p=0.590) | 0.310 (p=0.172) | -0.94  (p=0.685) | 0.008 (p=0.971) | 0.227 (p=0.323) |
| IL-8 (pg/ml) | 0.176 (p=0.458) | 0.015 (p=0.609) | 0.240 (p=0.309) | 0.155 (p=0.515) | 0.229 (p=0.331) | -0.064 (p=0.789) |
| HNE (AU/ml) | -0.044 (p=0.786) | 0.002 (p=0.772) | 0.108 (p=0.500) | -0.219  (p=0.169) | 0.141 (p=0.378) | -0.098 (p=0.554) |
| MUC5B (AU/ml) | 0.073 (p=0.669) | 0.080 (p=0.091) | 0.209 (p=0.214) | -0.223 (p=0.185) | 0.217 (p=0.197) | 0.144 (p=0.402) |
| MUC5AC(AU/ml) | 0.146 (p=0.387) | 0.048 (p=0.194) | 0.275 (p=0.100) | -0.388 (**p=0.018**) | 0.362 (**p=0.027**) | 0.289 (p=0.087) |

*Correlation with CPB-time, mechanical ventilation time, P/F ratio and ICU-LOS by Pearsons correlation coefficient. Correlation with type of surgery by ETA square correlation statistics. IL-8 = Interleukin 8; HNE = Human Neutrophil Elastase; MUC5B = Mucin 5B; MUC5AC = Mucin 5AC. CPB = cardiopulmonary bypass; ICU = intensive care unit *Simple: coronary artery bypass grafting with or without single valve surgery; Complex: all other surgery; AE/ml = Arbitrary Units/milliliter*

**References**

1. Richtlijn Bloedtransfusie beleid Nederlands: Federation of Medical Speciaslist, the Netherlands; [Available from: <https://richtlijnendatabase.nl/richtlijn/bloedtransfusiebeleid/startpagina_-_bloedtransfusiebeleid.html>.

2. Silbert BS, Santamaria JD, O'Brien JL, Blyth CM, Kelly WJ, Molnar RR. Early extubation following coronary artery bypass surgery: a prospective randomized controlled trial. The Fast Track Cardiac Care Team. Chest. 1998;113(6):1481-8.

3. Ramsay MA, Savege TM, Simpson BR, Goodwin R. Controlled sedation with alphaxalone-alphadolone. Br Med J. 1974;2(5920):656-9.

4. Dentener MA, Francot GJ, Hiemstra PS, Tool AT, Verhoeven AJ, Vandenabeele P, et al. Bactericidal/permeability-increasing protein release in whole blood ex vivo: strong induction by lipopolysaccharide and tumor necrosis factor-alpha. J Infect Dis. 1997;175(1):108-17.

5. Mertens TCJ, van der Does AM, Kistemaker LE, Ninaber DK, Taube C, Hiemstra PS. Cigarette smoke differentially affects IL-13-induced gene expression in human airway epithelial cells. Physiol Rep. 2017;5(13).
